# A combined immune and inflammatory indicator predict the prognosis of severe Pneumocystis jirovecii pneumonia patients: a 12-year, retrospective, observational cohort

**DOI:** 10.1186/s12890-024-03093-8

**Published:** 2024-06-18

**Authors:** Dong Wang, Lujia Guan, Xuyan Li, Zhaohui Tong

**Affiliations:** Department of Respiratory and Critical Care Medicine, Beijing Institute of Respiratory Medicine and Beijing Chao-Yang Hospital, Capital Medical University, 8 Gongren Tiyuchang Nanlua, Chaoyang District, Beijing, 100020 China

**Keywords:** Pneumocystis *jirovecii* pneumonia, Neutrophil-to-lymphocyte ratio, Mortality

## Abstract

Persistent inflammatory damage and suppressed immune function play a crucial role in the pathogenesis and progression of the pneumocystis *jirovecii* pneumonia (PjP). Therefore, we aimed to investigate the correlation between the combined immune and inflammatory indicator: the neutrophil-to-lymphocyte ratio (NLR) and prognosis of non-human immunodeficiency virus (non-HIV) PjP.

In the retrospective analysis conducted in ICUs at Beijing Chao-Yang Hospital, we examined data from 157 patients diagnosed with non-HIV PjP. Our findings reveal a concerning hospital mortality rate of 43.3%, with the 28-day mortality rate reaching 47.8%.

Through multivariable logistic and Cox regression analyses, we established a significant association between elevated NLR levels and hospital mortality (adjusted odd ratio, 1.025; 95% CI, 1.008-1.043; *p* = 0.004) or 28-day mortality (adjusted hazard ratio, 1.026; 95% CI, 1.008-1.045; *p* = 0.005). Specifically, patients with an NLR exceeding 20.3 demonstrated markedly lower overall survival rates, underscoring the biomarker's predictive value for both hospital and 28-day mortality.

In conclusion, non-HIV PjP patients in the ICU still have a high rate of mortality and a poor short-term prognosis after discharge. A high level of NLR was associated with an increased risk of hospital mortality and 28-day mortality.

**Supplementary Information:**

The online version contains supplementary material available at 10.1186/s12890-024-03093-8.

## Introduction

Pneumocystis *jirovecii* pneumonia (PjP) constitutes an opportunistic pulmonary fungal infection primarily affecting immunocompromised individuals. Since 1979, PjP has accounted for more than a quarter of opportunistic infections in the human immunodeficiency virus (HIV) population [1] and was once considered the most common opportunistic infection and the leading cause of death among those patients [2]. The widespread use of highly active antiretroviral therapy (HAART) and trimethoprim-sulfamethoxazole (TMP-SMX) has gradually reduced the incidence and mortality rates of PjP in the HIV-infected population [3]. With the increased use of immunosuppressants or cytotoxic agents in recent years, there has been a notable rise in PjP incidence among the non-HIV immunocompromised population not receiving prophylaxis [4]. Unlike their HIV-positive counterparts, individuals with non-HIV related immunosuppression often experience an acute onset of PjP, characterized by high fever and progressive respiratory distress that typically lasts for 4 to 7 days. This acute presentation can quickly lead to respiratory failure, necessitating intensive care unit (ICU) admission for over half of these patients [5] and resulting in mortality rates between 50 and 60% [5, 6]. This divergence in clinical presentation and outcomes underscores the need for studies that specifically address the characteristics and prognostic factors in non-HIV PjP patients.

Building on this context, it's recognized that the interplay between diminished immune function and an overactive inflammatory response is a key driver of pulmonary lesions in PjP [7]. Specifically, in those with compromised immune systems, a lack of lymphocytes impairs the clearance of Pneumocystis jirovecii (Pj), triggering an influx of neutrophils and a heightened inflammatory response. This exacerbation significantly impairs lung function, leading to hypoxemia and, in severe cases, respiratory failure [8, 9].

Identifying suitable biomarkers that comprehensively reflect the inflammatory and immune balance in PjP hosts is crucial for predicting patient outcomes. Previous studies have suggested that immune indices [9], and inflammatory factors [6, 10] are closely related to adverse hospital outcomes in non-HIV immunosuppressed patients with PjP. However, focusing solely on either immune or inflammatory markers appears insufficient to reveal the extent of host immune imbalance. The neutrophil-to-lymphocyte ratio (NLR) emerges as a pivotal biomarker, shedding light on the delicate balance between systemic inflammation and immune response [11]. While NLR has been extensively utilized in the prognosis of various diseases, including cancer [12], infectious diseases [13], and cardiovascular conditions [14], its prognostic value in non-HIV PjP patients remains unexplored.

Addressing this gap, our research endeavors to elucidate the clinical profile of PjP in the non-HIV immunocompromised cohort and to investigate the prognostic relevance of NLR within this group. Our objective is to furnish novel insights into the clinical prognostics and therapeutic management of PjP, potentially enhancing patient care and outcomes for this at-risk population.

## Methods

### Study design and patients

Between October 1, 2011, and July 31, 2023, our investigation involved 207 patients diagnosed with PjP following their discharge from various ICUs at Beijing Chao-Yang Hospital, encompassing the respiratory ICU, emergency ICU, and surgical ICU. This study was conducted in accordance with the principles of the Declaration of Helsinki and the experimental protocol for data involving human followed the Ethical Guidelines of the Ethics Committee of Beijing Chao-Yang Hospital, under the protocol number 2021-ke-192, and the need to obtain written informed consent was waived due to the retrospective nature.

### Inclusion criteria

Participants were eligible for this study if they: were 18 years of age or older; had a history of immunosuppression due to conditions such as malignancies (including hematological malignancies and solid tumors), bone marrow or solid organ transplantation, autoimmune diseases, or congenital immunodeficiency diseases; met the diagnostic criteria of acute respiratory distress syndrome [15].

Referencing prior research and a diagnostic guideline for non-HIV PjP [16], we defined the diagnosis of PjP using all of the following criteria:(a)Clinical symptoms like fever, cough, and dyspnea; (b)Chest imaging revealing bilateral diffuse ground glass opacities; (c)Positive identification of Pneumocystis cysts or trophozoites in respiratory specimens [qualified sputum samples, induced sputum, or bronchoalveolar lavage fluid (BALF)] via Grocott’s methenamine silver stain, or respiratory specimens testing positive for nucleic acid by metagenomics next-generation sequencing or polymerase chain reaction; (d)Elevated serum 1,3-β-glucan levels, with other fungal infections excluded [17].

A definitive diagnosis of PjP was established when a patient met the above clinical and diagnostic criteria (a and b, plus one of the conditions in c and d).

### Exclusion criteria

Patients were excluded if they: had positive serum HIV tests; were pregnant. For those with multiple admissions, the study considered only the first admission record to avoid duplication of data.

### Data collection

We systematically collected clinical data from the electronic medical record system at Beijing Chao-Yang Hospital. This comprehensive dataset included: demographic information (age, gender, and other relevant patient demographics), clinical characteristics (key features of each patient's clinical presentation and history), laboratory results (critical laboratory findings relevant to the prognosis and co-infections). The NLR was calculated based on the results of the first complete blood count performed within 24 h of patient admission. Treatment outcomes: this encompassed the length of ICU stay, the use and duration of invasive mechanical ventilation (IMV), and the use of veno-venous extracorporeal membrane oxygenation (VV-ECMO). Mortality Data: both hospital mortality and 28-day mortality rates were meticulously recorded to assess patient outcomes.

### Management

When non-invasive ventilation fails due to severe hypoxemia or excessive secretions, our protocol shifts to IMV using low tidal volumes and carefully adjusted positive end-expiratory pressure to optimize gas exchange and minimize lung injury [18]. If the PaO_2_/FiO_2_ ratio (PFR) is below 50 mmHg for over 30 min or if barotrauma occurs during IMV, VV-ECMO is used. All diagnosed cases in our study were managed according to the first-line treatment which involved administering trimethoprim at 15–20 mg/kg/day and sulfamethoxazole at 75–100 mg/kg/day for 21 days. For patients who cannot tolerate TMP-SMX, the combination of clindamycin and primaquine or caspofungin is the preferred alternative treatment [19].

### Statistical analysis

We performed all statistical analyses using the software R, version 4.0.2, and GraphPad Prism, version 9.0. We considered results to be statistically significant if the chance of the result occurring by random was less than 5% (*p*-value < 0.05).

For data that vary continuously, we described these using either the average value and the range around this average (mean and standard deviation, mean ± SD) for data that follow a normal distribution, or the middle value and the range between the lower and upper middle values [median and interquartile range, median(IQR)] for data that do not. For categories of data, we showed these as counts and percentages.

To compare groups for continuous data, we used either the One-Way ANOVA test (for data that's normally distributed) or the Mann-Whitney test (for data that's not). For categorical data, we used either the chi-squared test or Fisher’s exact test, depending on what was most suitable. We then identified factors that significantly affected the outcomes (*p*-value < 0.05 in univariate analysis) and important risk factors to include in a more detailed analysis using multivariate logistic and Cox regression models. We used receiver operating characteristic (ROC) curves to assess how well our predictive models could distinguish between different outcomes. To estimate survival probabilities over time for different groups, we used the Kaplan–Meier method and compared these groups using the log-rank test. We also looked into whether certain factors like age, gender, the PFR, and the use of IMV influenced the outcomes differently, performing subgroup analysis.

## Results

### Study population

**Fig. 1:**
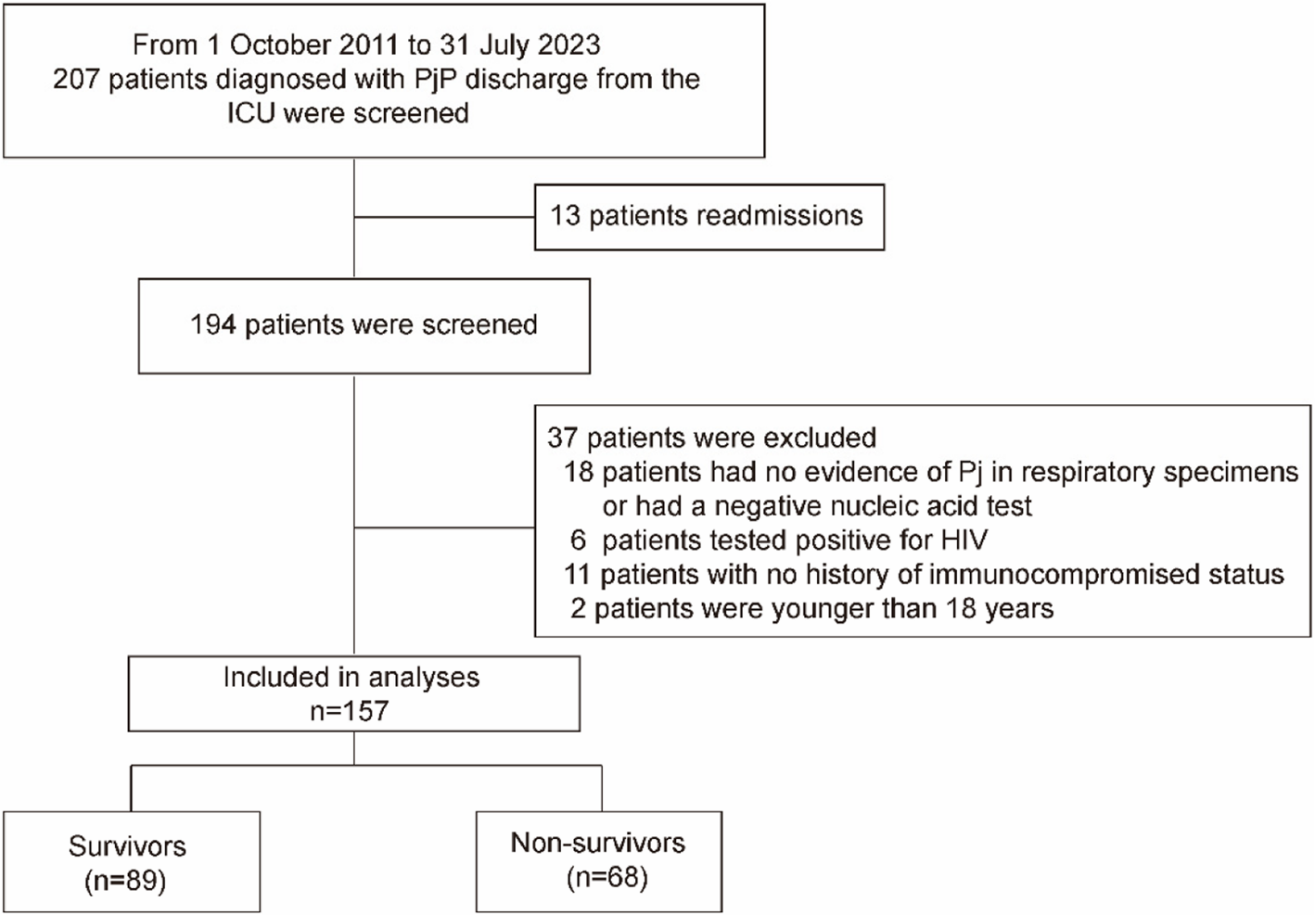
Study flow

Between October 2011 and July 2023, 207 patients diagnosed with PjP and discharged from the ICUs of Beijing Chao-Yang Hospital, were screened. Among them, 13 patients had multiple admissions, 6 patients were HIV-positive, 18 patients either had no evidence of Pj in respiratory specimens or had a negative nucleic acid test, 11 patients had no history of immunocompromised status. In addition, 2 patients were under 18 years of age, which led to their exclusion. Finally, 157 non-HIV PjP patients who met the inclusion criteria were analyzed (Fig. 1).

### Baseline characteristics

Table 1 shows the patients' baseline clinical characteristics. The mean (SD) age was 54.6 ± 15.0 years, 98/157 (62.4%) were male and median (IQR) body mass index (BMI) was 23.1(20.7-25.6) kg/m^2^. Solid organ transplantation was the initial cause of PjP, for 49 cases (31.2%), versus 41 (26.1%) with connective diseases and 30 (19.1%) with hematological diseases. Prior to the onset of PjP, 118 (75.2%) and 93 (59.2%) of the patients had been treated with corticosteroids and immunosuppressants including tacrolimus, mycophenolate mofetil, sirolimus, cyclophosphamide, methotrexate, and leflunomide. PjP was frequently associated with other pulmonary infections, with cytomegalovirus infection being the most common (68.2%), followed by bacterial infection (40.8%).

**Table 1:** Differences in baseline characteristics between survivors and non-survivors

Baseline characteristics	Whole cohort (*n* = 157)	Survivor (*n* = 89)	Non-survivors (*n* = 68)	*P*
Age, mean ± SD, year	54.6 ± 15.0	52.3 ± 15.4	57.6 ± 14.1	0.03
Male, n (%)	98(62.4)	55(61.8)	43(63.2)	0.80
BMI, median (IQR), kg/m^2^	23.1(20.7-25.6)	22.7(20.6-25.0)	23.9(21.2-26.2)	0.20
Time form onset symptoms to ICU, median (IQR), day	10(6-20)	10(3-22)	12(7-20)	0.83
Severity evaluation, median (IQR)				
APACHEII Score	12(8-15)	11(8-14)	13(9-15)	0.06
SOFA score	4(3-7)	4(3-6)	5(3-8)	0.05
Comorbidities, n (%)				
Hypertension	70(44.6)	45(50.6)	25(36.8)	0.08
Diabetes	31(19.7)	19(21.3)	12(17.6)	0.56
Coronary artery disease	13(8.3)	5(5.6)	8(11.8)	0.17
Chronic pulmonary disease	5(3.2)	1(1.1)	4(5.9)	0.09
Immunocompromised status, n (%)				
Solid organ transplantation				
Kidney transplantation	36(22.9)	24(27.0)	12(17.6)	0.17
Liver transplantation	13(8.3)	10(11.2)	3(4.4)	0.12
Cancer				
Lung cancer	1(0.6)	0	1(1.5)	0.25
Breast cancer	3(1.9)	3(3.4)	0	0.13
Esophageal cancer	3(1.9)	2(2.2)	1(1.5)	0.25
Thymoma	3(1.9)	2(2.2)	1(1.5)	0.25
Connective disease				
ANCA-associated vasculitis	6(3.8)	3(3.4)	3(4.4)	0.74
SLE	4(2.5)	3(3.4)	1(1.5)	0.45
Pemphigus	7(4.5)	4(4.5)	3(4.4)	0.98
Rheumatoid arthritis	9(5.7)	3(3.4)	6(8.8)	0.15
Dermatomyositis	7(4.5)	2(2.2)	5(7.4)	0.44
Autoimmune hemolytic anemia	4(2.5)	1(1.1)	3(4.4)	0.20
Others	4(2.5)	1(1.1)	3(4.4)	0.20
Hematological disease				
Non-Hodgkin's lymphoma	2(1.3)	1(1.1)	1(1.5)	0.85
Hodgkin's lymphoma	4(2.5)	1(1.1)	3(4.4)	0.20
Multiple myeloma	3(1.9)	1(1.1)	2(2.9)	0.41
Leukaemia	3(1.9)	3(3.4)	0	0.13
Hemophilia	3(1.9)	3(3.4)	0	0.13
Aplastic anemia	4(2.5)	2(2.2)	2(2.9)	0.79
Idiopathic thrombocytopenic purpura	11(7.0)	7(7.9)	4(5.9)	0.16
Idiopathic pulmonary fibrosis	13(8.3)	5(5.6)	8(11.8)	0.17
Nephrotic syndrome	18(11.5)	13(14.6)	5(7.4)	0.16
Immunosuppressive drugs before admission, n (%)				
Corticosteroids	118(75.2)	67(75.3)	51(75.0)	0.97
Immunosuppressant				
One immunosuppressant	45(28.6)	26(29.2)	19(27.9)	0.86
Two immunosuppressants	42(26.8)	25(28.1)	17(25.0)	0.67
Three immunosuppressants	6(3.8)	4(4.5)	2(2.9)	0.62
Vital signs, median (IQR)				
HR, rate/minute	94(80-108)	94(80-109)	95(79-105)	0.80
RR, rate/minute	25(20-29)	24(20-28)	26(22-30)	0.05
SBP, mmHg	123(112-138)	125(112-138)	120(112-137)	0.53
DBP, mmHg	75(63-82)	75(65-83)	73(61-81)	0.21
Laboratory results, median (IQR)				
WBC, median, 10^9^/L	8.4(5.5-10.8)	8.0(5.4-10.8)	8.5(5.7-10.8)	0.50
Neutrophil count, 10^9^/L	7.2(4.8-9.6)	7.2(4.8-9.3)	7.5(4.8-9.9)	0.47
Lymphocyte count, 10^9^/L	0.25(0.13-0.53)	0.29(0.18-0.55)	0.24(0.11-0.50)	0.22
NLR	24.4(13.3-40.4)	16.2(9.9-29.0)	33.7(23.3-60.9)	< 0.001
Monocyte, 10^9^/L	0.45(0.27-0.93)	0.56(0.30-1.13)	0.43(0.24-0.70)	0.60
Hemoglobin, g/L	108(93-120)	108(95-119)	102(57-125)	0.88
Platelet, 10^9^/L	166(107-234)	179(137-243)	136(71-205)	< 0.001
Albumin, g/L	27(24-31)	28(25-32)	26(22-29)	< 0.001
CRP, mg/L	12.5(8.7-15.9)	11.6(8.3-14.2)	14.4(10.4-17.3)	< 0.001
AST, U/L	20(10-32)	16(10-35)	31(12-31)	0.33
ALT, U/L	33(21-52)	32(22-51)	36(20-59)	0.87
BUN, mmol/L	8.3(5.8-14.3)	8.5(5.9-13.9)	7.6(5.6-15.3)	0.84
Crea, umol/L	78.3(54.4-147.8)	83.2(54.4-145.5)	77.2(54.1-157.0)	0.85
LDH, U/L	494(330-731)	406(283-551)	580(430-879)	< 0.001
TBIL, umol/L	8.9(6.1-14.4)	8.8(5.7-13.3)	9.3(6.1-15.1)	0.56
IBIL, umol/L	5.1(3.3-8.0)	5.2(3.2-7.9)	4.9(3.4-8.1)	0.82
Blood gas analysis				
PH	7.43(7.40-7.47)	7.43(7.40-7.46)	7.45(7.40-7.47)	0.34
P_a_CO_2_, mmHg	35.8(31.0-40.0)	35.0(30.6-38.0)	36.5(31.0-41.0)	0.34
P_a_O_2,_ mmHg	84(68-98)	86(73-99)	76(63-95)	0.03
PFR, mmHg	150.0(101.2-242.7)	190(120-268)	124(92-174)	< 0.001
Lymphocyte subsets				
CD3^+^ T cell, cell/ul	272(143-448)	306(162-489)	208(116-395)	0.02
CD4^+^ T cell, cell/ul	98(61-220)	139(82-251)	68(47-134)	< 0.001
CD8^+^ T cell, cell/ul	100(63-200)	125(75-251)	81(54-117)	< 0.001
Co-infections				
Bacterial infection	64(40.8)	43(48.3)	21(30.9)	0.03
Cytomegalovirus infection	107(68.2)	58(65.2)	49(72.1)	0.36
Fungal infection	58(36.9)	29(32.6)	29(42.6)	0.20
Outcomes				
Length of hospital	17(10-26)	12(8-24)	15(10-28)	< 0.001
IMV	90(57.3)	31(34.8)	59(86.8)	< 0.001
Duration of IMV	10(5.8-22)	8(5-23)	12(6-22)	0.62
VV-ECMO	18(11.5)	8(9.0)	10(14.7)	0.27

Data are presented as median (interquartile range), mean (standard deviation) or n (%); Other connective diseases included retroperitoneal fibrosis, sicca syndrome, adult-onset Still's disease, ankylosing spondylitis

*Abbreviations*: *SD* Standard deviation, *BMI* Body mass index, *IQR* Interquartile range, *ICU* Intensive care unit, *APACHEII* Acute physiology and chronic health evaluation II, *SOFA* Sepsis-related organ failure assessment, *ANCA* Anti-neutrophil cytoplasmic antibodies, *SLE* Systemic Lupus Erythematosus, *HR* Heart rate, *RR* Respiratory rate, *SBP* Systolic blood pressure, *DBP* Diastolic blood pressure, *WBC* White blood cell count, *NLR* Neutrophil–lymphocyte ratio, *CRP* C-reactive protein, *AST* Aspartate aminotransferase, *ALT* Alanine aminotransferase, *BUN* Blood urea nitrogen, *LDH* Lactate dehydrogenase, *TBIL* Total bilirubin, *IBIL* Indirect bilirubin, *PFR* PaO_2_/FiO_2_ ratio, *IMV* Invasive mechanical ventilation, *VV-ECMO* veno-venous extracorporeal membrane oxygenation

### Distribution of NLR in poor outcomes and subgroup patients

The NLR showed significant statistical differences between patients without IMV and those with IMV [18.1(9.8-28.0) vs 30.7(19.1-51.0), *p* < 0.001], between survivors and non-survivors [16.2(9.9-29.0) vs 33.7(23.3-60.9), *p* < 0.001], between 28-day mortality and non-28-day mortality [33.7(22.0-56.6) vs 16.2(10.0-28.9), *p* < 0.001], but no statistically significant difference between patients receiving VV-ECMO and those not receiving [34.3(15.0-64.8) vs 24.1(12.8-35.9), *p* = 0.153].The differences in NLR were not statistically significant in subgroups (*p* > 0.05) (shown in Supplementary Fig. 1, Supplementary Fig. 2 and Supplementary excel).

### Influence of NLR on the risk of hospital mortality and 28-day mortality

In multivariate logistic regression analysis using significant variables, together with clinically relevant risk factors, the NLR level was associated with both hospital mortality [adjusted odd ratio (OR), 1.025; 95% confidence interval (CI), 1.008-1.043; *p* = 0.004] and 28-day mortality [adjusted hazard ratio (HR), 1.026; 95% CI, 1.008-1.045; *p* = 0.005] (Table 2).

**Table 2:** Multivariable regression model results for hospital mortality and 28-day mortality

Patient characteristic	Hospital mortality		28-day mortality	
	Multivariable logistic regression		Multivariable Cox regression	
	OR (95% CI)	*P*^a^	HR (95% CI)	*P*^a^
NLR	1.025(1.008-1.043)	0.004	1.026(1.008-1.045)	0.005
Age	1.021(0.994-1.049)	0.122	1.015(0.990-1.041)	0.243
BMI	1.058(0.955-1.171)	0.280	1.081(0.964-1.186)	0.114
APACHE II score	1.006(0.926-1.093)	0.884	0.996(0.922-1.075)	0.914
SOFA score	1.160(0.997-1.349)	0.054	1.130(0.978-1.305)	0.097
Platelet	0.998(0.994-1.002)	0.354	0.997(0.993-1.001)	0.193
Albumin	0.919(0.844-1.001)	0.052	0.972(0.898-1.051)	0.476
LDH	1.001(0.999-1.002)	0.217	1.000(0.999-1.001)	0.911
PFR	0.998(0.994-1.002)	0.154	0.998(0.995-1.001)	0.188
CD4^+^ T cell	0.998(0.994-1.002)	0.268	0.997(0.994-1.001)	0.171

*Abbreviations*: *OR* Odd ratio, *HR* Hazard ratio, *CI* Confidence interval, *NLR* Neutrophil-lymphocyte ratio, *BMI* Body mass index, *APACHEII* Acute physiology and chronic health evaluation II, *SOFA* Sepsis-related organ failure assessment, *LDH* Lactate dehydrogenase, *PFR* PaO_2_/FiO_2_ ratio

^a^Adjusted by bacterial infection and cytomegalovirus infection

### Predictive power of NLR and other biomarkers for in-hospital mortality

**Fig. 2:**
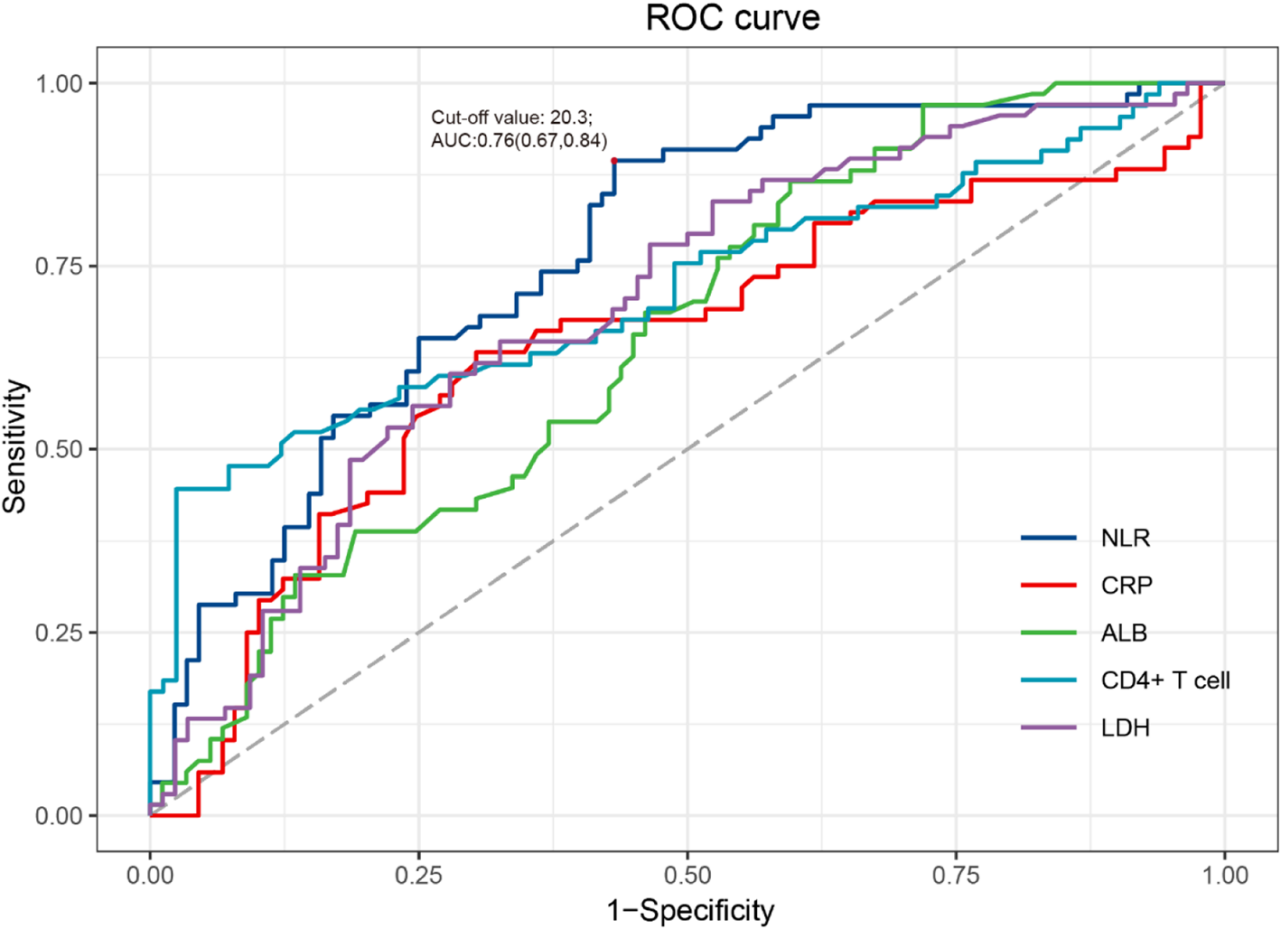
Receiver operating characteristic (ROC) curves for hospital mortality

The NLR had a maximum area under the Area Under Curve (AUC) (0.76; 95%CI, 0.67-0.84; *p* < 0.001), compared with C-reactive proteins (CRP) (ACU,0.63; 95%CI, 0.55-0.73; *p* = 0.002), albumin (ALB) (ACU,0.65; 95%CI, 0.57-0.74; *p* = 0.001), CD4^+^ T cell (AUC, 0.72; 0.63-0.80; *p* < 0.001), lactate dehydrogenase (LDH) (ACU,0.70; 95%CI, 0.61-0.78; *p* < 0.001) (Fig. 2 and Supplementary Table 1).

We established a cutoff value of 20.3 for NLR based on our analysis. Among patients with an NLR greater than or equal to 20.3, we found significantly higher proportions requiring invasive mechanical ventilation (75.4%), as well as higher in-hospital (88.5%) and 28-day mortality rates (77.6%). Kaplan-Meier analysis of survival in patients stratified for NLR < and ≥ 20.3 during 28 days (p log-rank test < 0.0001) (Supplementary Fig. 3).

### Subgroup analysis

Significant interaction relationships were observed among patients received IMV in hospital mortality (OR 1.060 [95% CI 1.021-1.110]; P for interaction < 0.001) and 28-day mortality (HR 1.008 [95% CI 1.001-1.015]; P for interaction = 0.023). (Supplementary Table 2).

### Differences in characteristics between the high NLR and low NLR groups

There was no statistical difference in baseline characteristics, underlying diseases, and vital signs between the two patient groups. Compared with the low NLR group, the high NLR group exhibited lower P_a_O_2_ [88.0(79.0-99.5) vs 78.0(63.5-96.0), *p* = 0.01]and PFR [205.0(119.3-278.9) vs 131.0(94.0-208.0), *p* = 0.01], and a higher proportion of IMV (37.9% vs 68.7%, *p* < 0.001).

## Discussion

Our study's exploration of non-HIV PjP in ICU-admitted patients uncovers pivotal insights into the prognosis of this immunosuppressed cohort. The utilization of IMV in 57.3% of patients and VV-ECMO in 11.5%, alongside observed hospital and 28-day mortality rates of 43.3% and 47.8% respectively, underscores the severity of PjP in these populations. Our analysis reveals that an elevated NLR significantly correlates with increased hospital and 28-day mortality, highlighting NLR above 20.3 as a critical marker of deteriorated survival prospects.

Recently, there has been a marked increase in the incidence of PjP among immunosuppressed individuals, particularly those with malignant tumors, undergoing organ or bone marrow transplantation, or suffering from inflammatory diseases [20]. Compared to HIV-infected PjP patients, non-HIV PjP patients often have more rapid disease progression and a poorer prognosis. Roux et al. [21] found that the non-HIV PjP patients presented mainly with non-specific symptoms such as fever, cough, and dyspnea. Their disease progression was significantly more acute, with a median onset of 5 days compared to 21 days in the HIV-infected PjP patients (*p* < 0.05). They also had more severe hypoxemia, were more likely to require ICU admission (50% vs. 35%, *p* < 0.05), and were more likely to require both non-invasive ventilation (16% vs. 8%, *p* < 0.05) and IMV (30% vs. 11%, *p* < 0.05). These findings resonate with our previous research [22]. In the present study, 57.3% availed of IMV, and the observed hospital mortality rate was 43.3%. In summary, non-HIV PjP patients are at a heightened risk for unfavorable outcomes, such as mortality, and reliance on IMV or VV-ECMO. Recognizing the prognostic markers for this group is crucial for clinicians to make well-informed therapeutic decisions.

While immune markers [23] and inflammatory factors [6, 10] are associated with adverse outcomes in patients with PjP who are non-HIV immunosuppressed, the NLR offers superior prognostic accuracy for mortality. Previous studies have highlighted various biomarkers, such as LDH, CRP, and albumin, as predictors of patient outcomes in non-HIV immunosuppressed PjP patients. For instance, Schmidt et al. found that LDH levels at admission were associated with adverse outcomes during hospitalization in a mixed population of HIV-infected and non-HIV immunosuppressed PjP patients [6]. In addition, compared with HIV-infected individuals who have not undergone HAART, those who initiate HAART treatment exhibit a decrease in serum LDH levels, which may indicate a reduction in the extent of lung damage [24]. Similarly, high CRP levels have been correlated with adverse outcomes, underscoring its role in reflecting the inflammatory status [25]. It is currently believed that hypoproteinemia in non-HIV immunosuppressed PjP patients results from both acute inflammation caused by PjP and chronic inflammation due to underlying diseases. Akahane et al. found that survivors of non-HIV PjP had significantly higher albumin levels than non-survivors [26]. Despite these associations, NLR stands out by providing a more direct and potent indicator of systemic inflammation and immune response, making it a more reliable predictor of mortality in PjP patients.

In HIV patients, the HIV virus targets CD4^+^ T lymphocytes, leading to their progressive decline, which significantly increases the risk of PjP [27]. This reduction in CD4^+^ T lymphocytes is a central feature of HIV progression and a critical marker for immunosuppression in this population. In contrast, non-HIV immunosuppressed patients face a broader spectrum of immunosuppressive influences due to various medications, impacting numerous immune pathways and biological processes. Our previous study showed significant suppression of B lymphocyte immune-related gene expression with glucocorticoid-induced Pneumocystis pneumonia [28]. Rong et al. further demonstrated an increased burden of Pneumocystis *carinii *(Pc) and delayed clearance of Pc in the lungs of mice with a lack of B-cell function [29]. We propose that NLR might offer a more universally applicable prognostic marker across different immunocompromised states, including but not limited to HIV.

In our analysis, patients with high NLR exhibited poorer oxygenation and a greater need for IMV compared to those with low NLR, indicating that a high NLR may reflect severe lung injury. A detailed analysis [30] of the BALF from 166 non-HIV PjP patients showed that a neutrophil-dominant presence was rare in cases of mild to moderate non-HIV PjP. However, it was present in about one-third of the more severe cases. Interestingly, the proportion of neutrophils in BALF was significantly associated with 30-day (OR: 1.02, 95%CI: 1.01-1.03) and 60-day all-cause mortality (OR: 1.02, 95%CI: 1.01-1.04). At the same time, we also found that compared with patients with low NLR, patients with high NLR showed a downward trend in T cell subsets, but the difference was not statistically significant. This finding suggests that low T cell counts do not necessarily correlate with high NLR. Factors beyond simple lymphocyte depletion, such as acute phase reactions or other pathophysiological mechanisms, may influence NLR values in this specific patient cohort. Therefore, while T cell counts are critical indicators of immune status, NLR may provide a more comprehensive understanding of the complex interplay between immune response and systemic inflammatory in patients with PjP.

Although our retrospective analysis provides initial insights into the use of the NLR, comprehensive prospective studies are required to fully exploit its potential as a clinical tool. Future research should focus on monitoring changes in NLR and correlating these with patient progression, evaluating the generalizability and adaptability of NLR across various medical settings, and determining optimal thresholds to guide or escalate clinical interventions. These efforts are essential for integrating NLR into routine clinical practice effectively.

Our study has several limitations. Firstly, this study is a single-center study, and the immunosuppressed population is dominated by autoimmune diseases and solid organ transplantation, which may limit the generalization of the results; Secondly, NLR might be influenced by the diverse clinical backgrounds or medications of the patients in our cohort, and a larger cohort is needed to reduce heterogeneity; Thirdly, we evaluated the patients' primary clinical outcomes including ICU deaths, but did not examine the patients' long term survivorship.

## Conclusion

In conclusion, non-HIV PjP patients in the ICU still have a high rate of mortality and a poor short-term prognosis after discharge. A high level of NLR was associated with an increased risk of hospital mortality and 28-day mortality.

### Supplementary Information

Supplementary Material 1.

Supplementary Material 2.

## Data Availability

The datasets used and/or analyzed during the current study are available from the corresponding author on reasonable requests.

## Abbreviations

PjP: Pneumocystis *jirovecii* pneumonia
HIV: Human immunodeficiency virus
HAART: Highly active antiretroviral therapy
TMP-SMX: Trimethoprim-sulfamethoxazole
ICU: Intensive care unit
Pj: Pneumocystis *jirovecii*
NLR: Neutrophil to lymphocyte ratio
BALF: Bronchoalveolar lavage fluid
IMV: Invasive mechanical ventilation
VV-ECMO: Veno-venous extracorporeal membrane oxygenation
PFR: PaO_2_/FiO_2_ ratio
SD: Standard deviation
IQR: Interquartile range
ROC: Receiver operating characteristic
BMI: Body mass index
OR: Odd ratio
CI: Confidence interval
HR: Hazard ratio
AUC: Area under curve
CRP: C-reactive proteins
ALB: Albumin
LDH: Lactate dehydrogenase
Pc: Pneumocystis carinii
