# Supplementary material for: A combined immune and inflammatory indicator predict the prognosis of severe Pneumocystis jirovecii pneumonia patients: a 12-year, retrospective, observational cohort
Source: BMC Pulm Med. 2024 Jun 18;24:285. doi: 10.1186/s12890-024-03093-8 (PMC11186281; doi:10.1186/s12890-024-03093-8)
Supplement: Supplementary file 1 — Supplementary Material 1. [file 12890_2024_3093_MOESM1_ESM.docx]

**Supplementary material to :** “A combined immune and inflammatory indicator predict the prognosis of severe Pneumocystis jirovecii pneumonia patients：a 12-year, retrospective, observational cohort”

**CONTENTS**

**Supplementary Tables:**

Supplementary *Table 1*

Supplementary *Table 2*

Supplementaty *Table 3*

**Supplementary Figures:**

Supplementary *Figure 1*

Supplementary *Figure 2*

Supplementary *Figure 3*

**Supplementary Table 1: AUC (with 95%CI) for the prediction of hospital mortality for different biomarkers**

| Biomarkers | AUC | 95% CI | *P* |
| --- | --- | --- | --- |
| NLR | 0.76 | 0.67-0.84 | <0.001 |
| CRP | 0.63 | 0.55-0.73 | 0.002 |
| ALB | 0.65 | 0.57-0.74 | 0.001 |
| CD4^+^T cell | 0.72 | 0.63-0.80 | <0.001 |
| LDH | 0.70 | 0.61-0.78 | <0.001 |

Abbreviations: AUC, area under curve; CI, confidence interval; NLR, neutrophil-lymphocyte ratio; CRP, C-reactive protein; ALB, albumin; LDH, lactate dehydrogenase.

**Supplementary Table 2: Subgroup analysis for the association of NLR with hospital mortality and 28-day mortality**

| Variables | No. of Patients | Hospital mortality | *P* | No. of Patients | 28-day mortality | *P* |
| --- | --- | --- | --- | --- | --- | --- |
|  |  | OR (95%CI) |  |  | HR (95%CI) |  |
| Age |  |  | 0.848 |  |  | 0.184 |
| ＜65 years | 43/114 | 1.018(1.001-1.035) |  | 50/114 | 1.007(1.000-1.014) |  |
| ≥65 years | 25/43 | 1.185(1.000-1.405) |  | 25/43 | 1.006(0.992-1.021) |  |
| Sex |  |  | 0.699 |  |  | 0.758 |
| Male | 43/98 | 1.027(1.006-1.050) |  | 44/98 | 1.009(1.001-1.016) |  |
| Female | 25/59 | 1.027(0.994-1.062) |  | 31/59 | 1.004(0.994-1.015) |  |
| BMI |  |  | 0.791 |  |  | 0.935 |
| <25 | 46/111 | 1.030(1.004-1.058) |  | 50/111 | 1.004(0.995-1.014) |  |
| ≥25 | 22/46 | 1.029(1.001-1.057) |  | 25/46 | 1.017(1.004-1.029) |  |
| IMV |  |  | 0.000 |  |  | 0.023 |
| Yes | 59/90 | 1.060(1.021-1.110) |  | 63/90 | 1.008(1.001-1.015) |  |
| No | 9/67 | 1.011(0.978-1.044) |  | 12/67 | 0.998(0.977-1.019) |  |
| PFR |  |  | 0.671 |  |  | 0.919 |
| <150 mmHg | 45/78 | 1.029(01.006-1.054) |  |  | 1.009(1.002-1.015) |  |
| ≥150 mmHg | 23/79 | 1.029(0.996-1.062) |  |  | 1.006(0.993-1.020) |  |
| Bacterial infection |  |  | 0.240 |  |  | 0.195 |
| Yes | 21/64 | 1.029(0.999-1.059) |  | 28/64 | 1.004(0.993-1.014) |  |
| No | 47/93 | 1.024(1.001-1.048) |  | 47/93 | 1.009(1.001-1.016) |  |

Abbreviations: OR, odd ratio; CI, confidence interval; BMI, body mass index; HR, hazard ratio; IMV, invasive mechanical ventilation

**Supplementary Table 3:** **Differences in characteristics between NLR subgroup**

| Baseline characteristics | NLR≥20.3  (n=99) | NLR<20.3  (n=58) | *P* |
| --- | --- | --- | --- |
| Age, mean±SD, year | 55.4±14.5 | 53.1±15.9 | 0.34 |
| Male, n (%) | 59(59.6) | 39(67.2) | 0.34 |
| BMI, median (IQR), kg/m^2^ | 23.2(21.1-26.0) | 22.8(20.2-25.2) | 0.66 |
| Time form onset symptoms to ICU  , median (IQR), day | 10(7-17) | 12(6-30) | 0.29 |
| Severity evaluation, median (IQR) |  |  |  |
| APACHEII Score | 12(9-15) | 9(8-14) | 0.08 |
| SOFA score | 4(3-7) | 4(3-7) | 0.63 |
| Comorbidities, n (%) |  |  |  |
| Hypertension | 45(45.5) | 25(43.1) | 0.78 |
| Diabetes | 21(21.2) | 10(17.5) | 0.55 |
| Coronary artery disease | 10(10.1) | 3(5.2) | 0.28 |
| Chronic pulmonary disease | 4(4.0) | 1(1.7) | 0.43 |
| Immunocompromised status, n (%) |  |  |  |
| Solid organ transplantation |  |  |  |
| Kidney transplantation | 18(18.2) | 18(31.0) | 0.06 |
| Liver transplantation | 6(6.1) | 7(12.1) | 0.19 |
| Cancer |  |  |  |
| Lung cancer | 1(1.0) | 0 | 0.25 |
| Breast cancer | 1(1.0) | 2(3.4) | 0.44 |
| Esophageal cancer | 2(2.2) | 1(1.7) | 0.25 |
| Thymoma | 1(1.0) | 2(3.4) | 0.19 |
| Connective disease |  |  |  |
| ANCA-associated vasculitis | 5(5.1) | 1(1.7) | 0.29 |
| SLE | 3(3.0) | 1(1.7) | 0.62 |
| Pemphigus | 3(3.0) | 4(6.9) | 0.26 |
| Rheumatoid arthritis | 7(7.1) | 2(3.4) | 0.35 |
| Dermatomyositis | 5(5.1) | 2(3.4) | 0.43 |
| Autoimmune hemolytic anemia | 3(3.0) | 1(1.7) | 0.62 |
| Others | 2(2.0) | 2(3.4) | 0.58 |
| Hematological disease |  |  |  |
| Non-Hodgkin's lymphoma | 1(1.0) | 1(1.7) | 0.70 |
| Hodgkin's lymphoma | 3(3.0) | 1(1.7) | 0.62 |
| Multiple myeloma | 3(3.0) | 0 | 0.18 |
| Leukaemia | 2(2.0) | 1(1.7) | 0.90 |
| Hemophilia | 1(1.0) | 2(3.4) | 0.28 |
| Aplastic anemia | 3(3.0) | 1(1.7) | 0.62 |
| Idiopathic thrombocytopenic purpura | 9(9.1) | 2(3.4) | 0.18 |
| Idiopathic pulmonary fibrosis | 9(9.1) | 4(6.9) | 0.63 |
| Nephrotic syndrome | 11(11.1) | 7(12.1) | 0.86 |
| Immunosuppressive drugs before admission, n (%) |  |  |  |
| Corticosteroids | 76(76.8) | 42(72.4) | 0.54 |
| Immunosuppressant |  |  |  |
| One immunosuppressant | 26(26.3) | 19(32.8) | 0.39 |
| Two immunosuppressants | 27(27.3) | 15(25.9) | 0.85 |
| Three immunosuppressants | 5(5.1) | 1(1.7) | 0.29 |
| Vital signs, median (IQR) |  |  |  |
| HR, rate/minute | 95(78-107) | 93(82-109) | 0.55 |
| RR, rate/minute | 25(22-30) | 24(20-29) | 0.18 |
| SBP, mmHg | 124(115-138) | 122(109-139) | 0.52 |
| DBP, mmHg | 74(62-83) | 76(65-82) | 0.50 |
| Laboratory results, median (IQR) |  |  |  |
| WBC, median, 10^9^/L | 8.6(6.1-10.8) | 7.7(4.9-10.8) | 0.20 |
| Neutrophil count, 10^9^/L | 7.5(5.3-9.9) | 6.2(4.3-9.2) | 0.09 |
| Lymphocyte count, 10^9^/L | 0.2(0.1-0.4) | 0.5(0.3-0.7) | <0.001 |
| Monocyte, 10^9^/L | 0.4(0.3-0.8) | 0.6(0.3-1.2) | 0.20 |
| Hemoglobin, g/L | 105(88-120) | 109(95-120) | 0.46 |
| Platelet, 10^9^/L | 167(88-229) | 166(120-236) | 0.33 |
| Albumin, g/L | 27.4(24.2-33.1) | 27.1(23.5-30.0) | 0.16 |
| AST, U/L | 20(10-32) | 19(10-31) | 0.81 |
| ALT, U/L | 31(20-51) | 36(25-54) | 0.51 |
| BUN, mmol/L | 7.6(5.9-14.2) | 9.2(5.8-15.1) | 0.58 |
| Crea, umol/L | 75.5(54.3-146.3) | 83.9(55.9-157.2) | 0.48 |
| LDH, U/L | 539(351-804) | 412(283-571) | 0.01 |
| TBIL, umol/L | 8.9(6.1-14.4) | 9.2(6.0-14.4) | 0.85 |
| IBIL, umol/L | 4.7(3.3-8.1) | 5.2(3.3-8.0) | 0.78 |
| Blood gas analysis |  |  |  |
| PH | 7.44(7.40-7.47) | 7.43(7.40-7.46) | 0.53 |
| P_a_CO_2_, mmHg | 35.3(30.3-40.0) | 36.0(32.0-39.7) | 0.72 |
| P_a_O_2,_ mmHg | 78.0(63.5-96.0) | 88.0(79.0-99.5) | 0.01 |
| PFR, mmHg | 131.0(94.0-208.0) | 205.0(119.3-278.9) | 0.01 |
| Lymphocyte subsets |  |  |  |
| CD3^+^T cell, cell/ul | 237(135-467) | 286(149-428) | 0.29 |
| CD4^+^T cell, cell/ul | 94(49-193) | 114(75-234) | 0.07 |
| CD8^+^T cell, cell/ul | 97(55-173) | 117(70-251) | 0.05 |
| Co-infections |  |  |  |
| Bacterial infection | 35(35.4) | 29(50.0) | 0.07 |
| Cytomegalovirus infection | 69(69.7) | 38(65.5) | 0.59 |
| Fungal infection | 40(40.4) | 18(31.0) | 0.24 |
| Outcomes |  |  |  |
| Length of hospital | 20(11-29) | 19(12-50) | 0.93 |
| IMV | 68(68.7) | 22(37.9) | <0.001 |
| Duration of IMV | 13(6-23) | 8(5-12) | 0.35 |
| VV-ECMO | 11(11.1) | 7(12.1) | 0.86 |

Note: Data are presented as median (interquartile range), mean (standard deviation) or n (%); Other connective diseases included retroperitoneal fibrosis, sicca syndrome, adult-onset Still's disease, ankylosing spondylitis

Abbreviations: SD, standard deviation; BMI, body mass index; IQR, interquartile range; ICU, intensive care unit; APACHEII, acute physiology and chronic health evaluation II; SOFA, sepsis-related organ failure assessment; ANCA, anti-neutrophil cytoplasmic antibodies; SLE, systemic Lupus Erythematosus; HR, heart rate; RR, respiratory rate; SBP, Systolic blood pressure; DBP, Diastolic blood pressure; WBC, white blood cell count; NLR, neutrophil-lymphocyte ratio; AST, aspartate aminotransferase; ALT, alanine aminotransferase; BUN, blood urea nitrogen; LDH, lactate dehydrogenase; TBIL, total bilirubin; IBIL, indirect bilirubin; PFR, PaO_2_/FiO_2_ ratio; IMV, invasive mechanical ventilation; VV-ECMO, veno-venous extracorporeal membrane oxygenation.


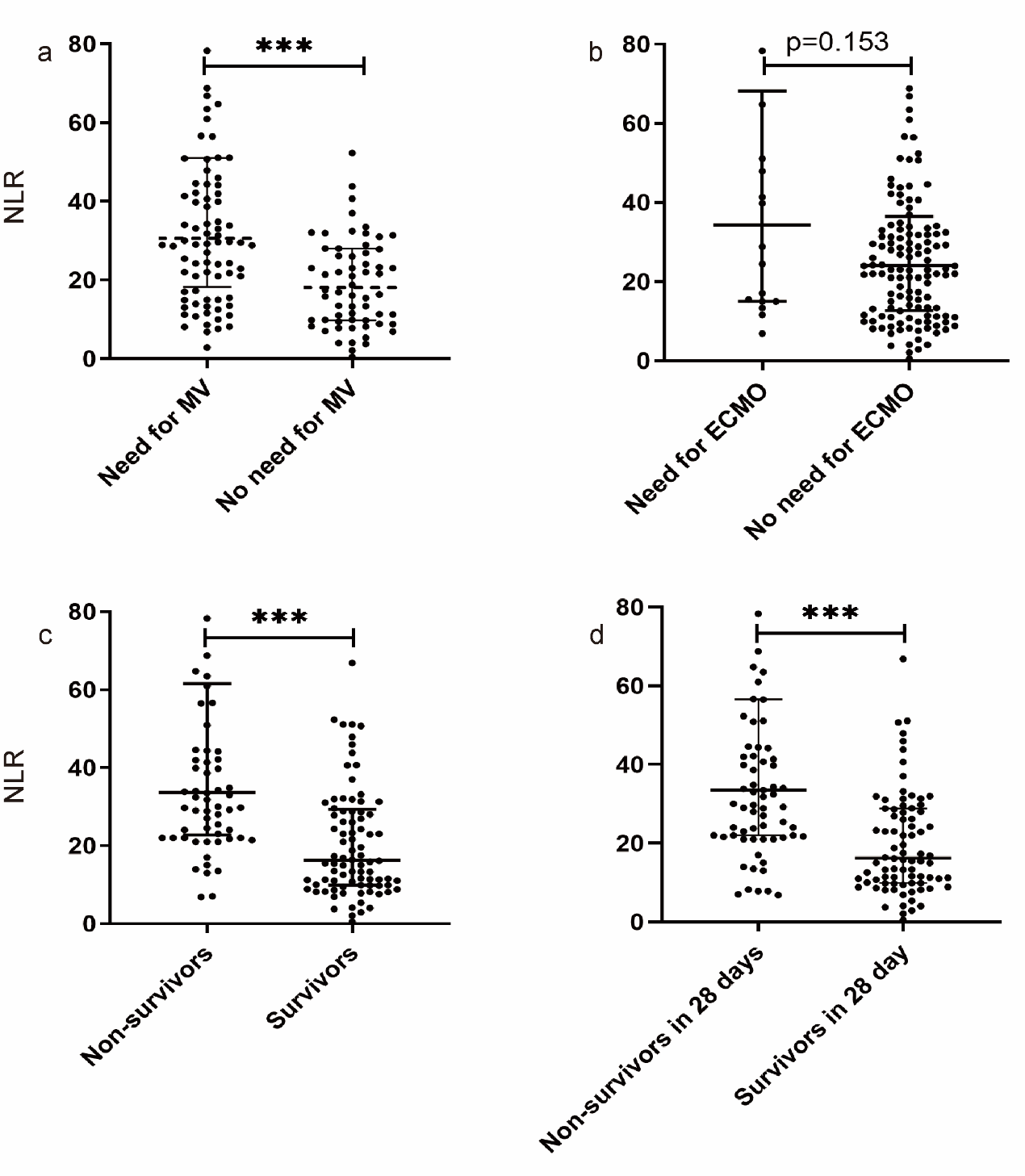


**Supplementary Figure 1: Distribution of NLR in poor outcomes.** Panel a shows the distribution of NLR in patients with and without MV; Panel b shows the distribution of NLR in patients with and without ECMO; Panel c shows the distribution of NLR in survived and non-survivors; Panel d shows the distribution of NLR in patients between 28-day mortality and non 28-day mortality.

Note:***,p<0.001


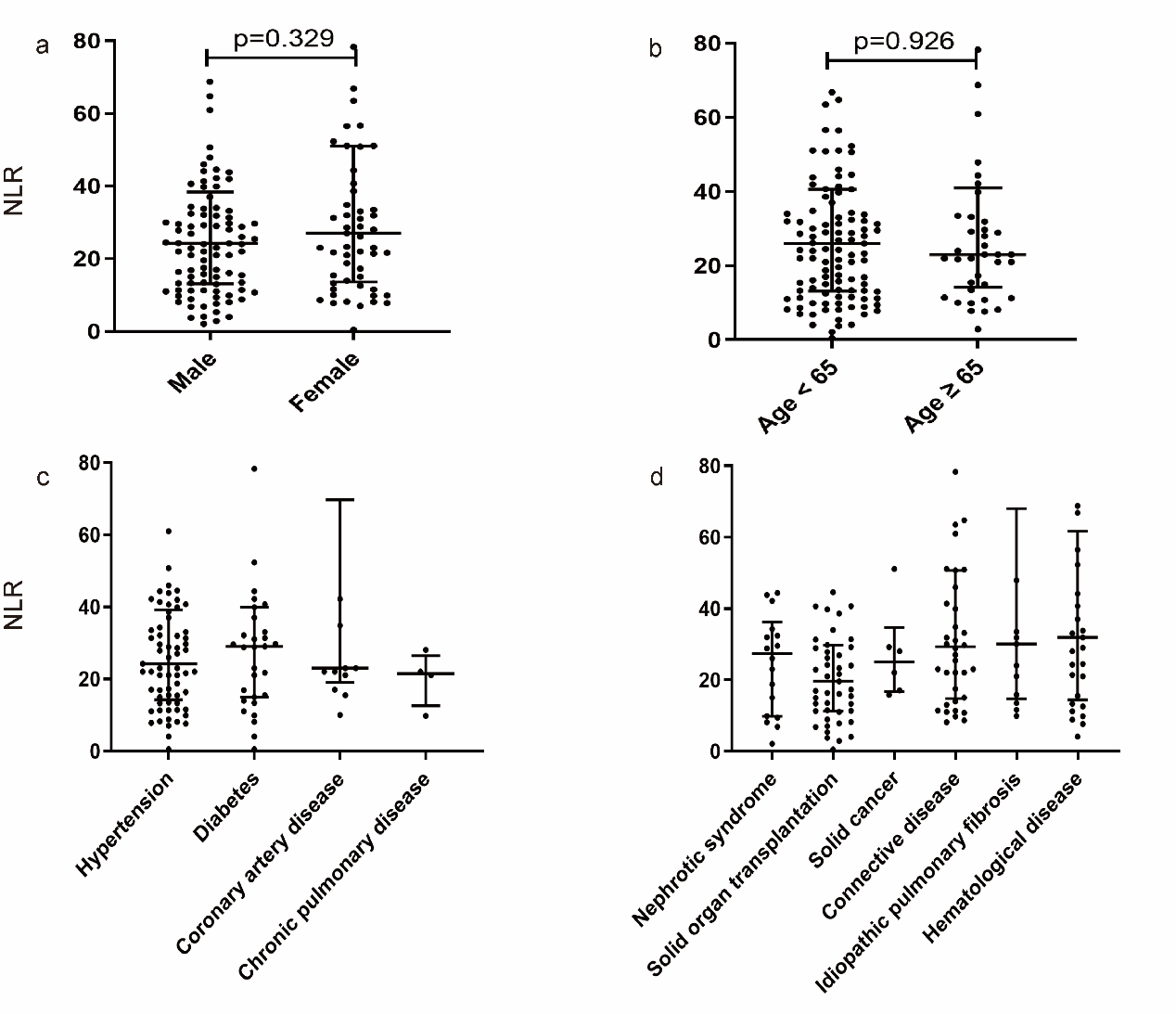


**Supplementary Figure 2: Distribution of NLR in different populations.**

Panel a shows the distribution of NLR in male and female; Panel b shows the distribution of NLR in patients older and younger ; Panel c shows the distribution of NLR in different comorbidities; Panel d shows the distribution of NLR in different immunocompromised status.


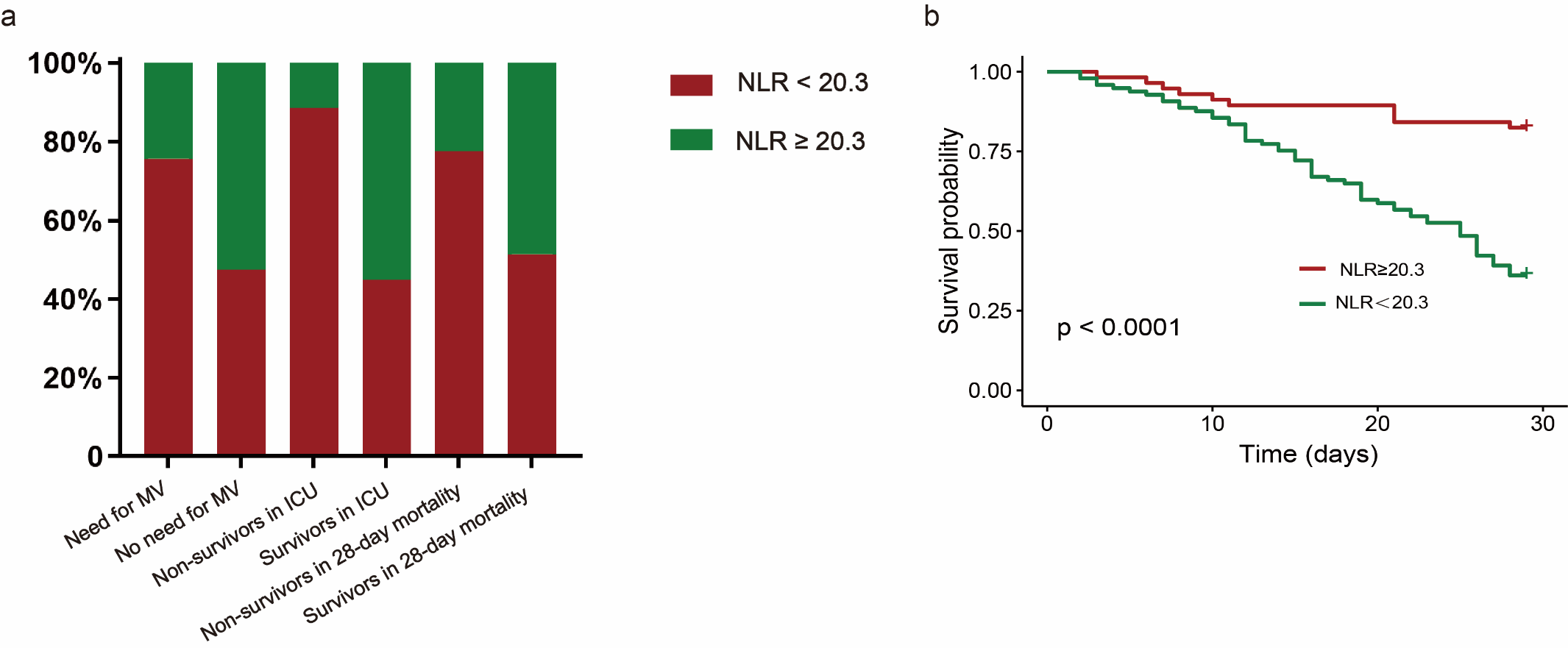
**Supplementary Figure 3: Description of outcomes and Kaplan–Meier curve based on cut-off value.** Panel a shows the proportions of NLR subgroups in different outcomes; Panel b shows Kaplan–Meier analysis of survival in patients with different NLR subgroups
